# Treatment with Methotrexate in Infants and Toddlers with Atopic Dermatitis: A Retrospective Multi-Center Study

**DOI:** 10.3390/jcm12165409

**Published:** 2023-08-20

**Authors:** Jen A. Barak Levitt, Sima Alemi, Ayelet Ollech, Shiran Reiss-Huss, Mohammad Sah, Yael Renert-Yuval, Rivka Friedland, Shoshana Greenberger, Eran Cohen Barak

**Affiliations:** 1 Department of Dermatology, Emek Medical Center, Afula 1834111, Israel; 2 Department of Dermatology, Pediatric Dermatology Service, Sheba Medical Center, Ramat-Gan 5265601, Israel; 3 Faculty of Medicine, Tel Aviv University, Tel Aviv 6997801, Israel; 4 Pediatric Dermatology Unit, Schneider Children’s Medical Center of Israel, Petah Tikva 4920235, Israel; 5 The Ruth and Bruce Rappaport Faculty of Medicine, Technion, Haifa 3200003, Israel

**Keywords:** atopic dermatitis (AD), pediatric patients, toddlers, infants, methotrexate

## Abstract

Atopic dermatitis (AD) is a chronic inflammatory skin disease affecting up to 20% of children. Methotrexate (MTX) is used off-label as a systemic treatment for AD patients unresponsive to topical therapies, but limited data exist regarding its safety and efficacy in children, especially in those < 4 years old. To further investigate MTX in younger patients, we screened the medical records of three referral centers between 2016 and 2022 and identified 28 infants and toddlers < 4 years old with AD treated with MTX. Mean age upon MTX initiation was 2.7 ± 1.2 years and mean investigator global assessment (IGA) score was 3.78 ± 0.4. Median duration of MTX treatment was five months. Following 12 and 24 weeks of MTX treatment, the response rate was 50% and IGA 0/1 was achieved in 14.2% and 21.4% of patients, respectively. Most treatment cessations were attributed to a lack of efficacy or parental concern. Although adverse events were reported in 57.1% of patients, MTX was discontinued due to such adverse events only in two patients (7.1%). Taken together, MTX demonstrated a high safety profile in AD patients <4 years old. MTX efficacy was moderate and presumably underestimated by parents who opted for premature treatment cessation due to concerns associated with an immunomodulatory drug.

## 1. Introduction

Atopic dermatitis (AD) is a common, chronic relapsing inflammatory skin disease that affects approximately 2–5% [1] of the adult population and 5–20% [2] of the pediatric population in the Western world. Its impact on pediatric patients and their families is significant and correlated with disease severity [3]. Early onset AD is common, with approximately 60% developing symptoms within the first year of life and 90% by age five [4]. Early onset AD is characterized by the simultaneous presence of food and inhalant allergies and a higher risk of developing asthma as part of the “atopic march” [5]. Among those affected early in life, about 20% experience persistent symptoms [4] and a more severe disease [6,7]. In Israel specifically, 25% of AD patients under the age of six suffer of severe disease [8].

Treatment of pediatric patients with atopic dermatitis requires a multifaceted approach. This includes identifying and avoiding provocation factors, maintaining a consistent skincare routine with emollients, and implementing dietary interventions in cases of food allergies. Topical anti-inflammatory therapy, including glucocorticosteroids and calcineurin inhibitors, is the mainstay of treatment for mild–moderate disease [9,10].

Toddlers with moderate–severe AD display a unique clinical challenge: widespread, chronic use of topical corticosteroids is often not sufficient and may be associated with adverse effects (primarily at the site of use). The use of phototherapy is limited in this population as young children cannot undergo treatment without a caregiver, and treatment with immunosuppressive agents (cyclosporine A, azathioprine, mycophenolate mofetil) have significant potential systemic adverse events, especially when used long-term.

Dupilumab is a monoclonal antibody directed against the IL-4 receptor [10], which was recently approved in infants and toddlers with AD and showed excellent efficacy and safety profile [11,12]. However, payers still define previous immunosuppressive therapy as a prerequisite requirement for biological therapy [8,13]; thus, conventional systemic therapy is still required in these patients.

Methotrexate (MTX) is an optional off-label systemic agent for treating AD patients who do not respond to topical regimens and/or phototherapy [14]. The active mechanism of methotrexate in patients with AD is not fully understood, but it is known to have anti-inflammatory properties and to also reduce allergen-specific T-cell activity [15]. Although potential adverse events include gastrointestinal disturbance, liver function abnormalities, and bone marrow suppression, MTX is generally well tolerated and considered safe for long-term use in pediatric patients with psoriasis and rheumatic diseases [16,17,18]. Data supporting the use of MTX for treating AD in toddlers (0–4 years old) are scarce compared to studies in adults, adolescents, and older children (5–12 years old) [19,20].

Herein, we aimed to explore the clinical profile of pediatric patients under the age of four years old treated with methotrexate, as well as the efficacy and safety of treatment after 12 and 24 weeks of therapy. 

## 2. Materials and Methods

Study Design: This study employed a retrospective design. Data were collected between 2016 and 2022 from three medical centers in Israel: Emek Medical Center, Sheba Medical Center, and Schneider Medical Center. Ethical approval was obtained from the review boards of all three institutions.

Participants: The cohort consists of a total of 28 patients who met the inclusion criteria:

Age under four years, diagnosis of AD by certified dermatologists based on the Hannifin and Rajika AD diagnostic criteria [21], and methotrexate treatment for AD for at least 8 weeks. Patients’ parents gave their informed consent to use MTX as an off-label drug for AD. All patients were supplemented with folic acid with the regimen of 400 mcg 5 days a week, excluding the day of MTX administration and the day before it. Any patient records that did not meet the inclusion criteria or that had partial information were excluded from the analysis.

Data and Data Collection: Data collection was performed by experienced dermatologists at each medical center. The medical records of the eligible patients were reviewed to extract relevant information for the study. Demographic features as well as disease variables were collected, including age, sex, duration, and severity of disease. The latter was assessed by IGA score (investigator global assessment) [22]. Past treatments and personal and family history of atopy were also collected. Treatment variables included age at onset of treatment, treatment duration, IGA score after 12 and 24 weeks of treatment, adverse effects, reasons for treatment cessation, frequency of laboratory monitoring, and next planned treatment line. A partial response was defined as a 50% reduction in the initial IGA score. A complete/almost complete response was defined as the achievement of an IGA score of 0/1. The response rate was defined as the fraction of patients who achieved partial or complete response of all treated patients.

Data Analysis: Descriptive statistics were employed to summarize the demographic and clinical characteristics of the study population. Categorical variables were presented as frequencies and percentages, while continuous variables were reported as means with standard deviations or medians with interquartile ranges, depending on their distribution. The Wilcoxon test was used to compare IGA before and after treatment. Data analysis was performed using Microsoft Excel. 

## 3. Results

Demographic and Clinical Characteristics. A total of 28 patients participated in the study. Eleven (39.3%) were female. The median age at disease onset was 0.4 years (IQR = 0.9). Patient demographic and clinical characteristics are presented in Table 1. AD severity prior to MTX initiation was evaluated using the IGA scale, with a mean score of 3.78 (SD = 0.4). The average IgE level was 554 IU/mL (IQR = 1502.2).

**Figure 1:**
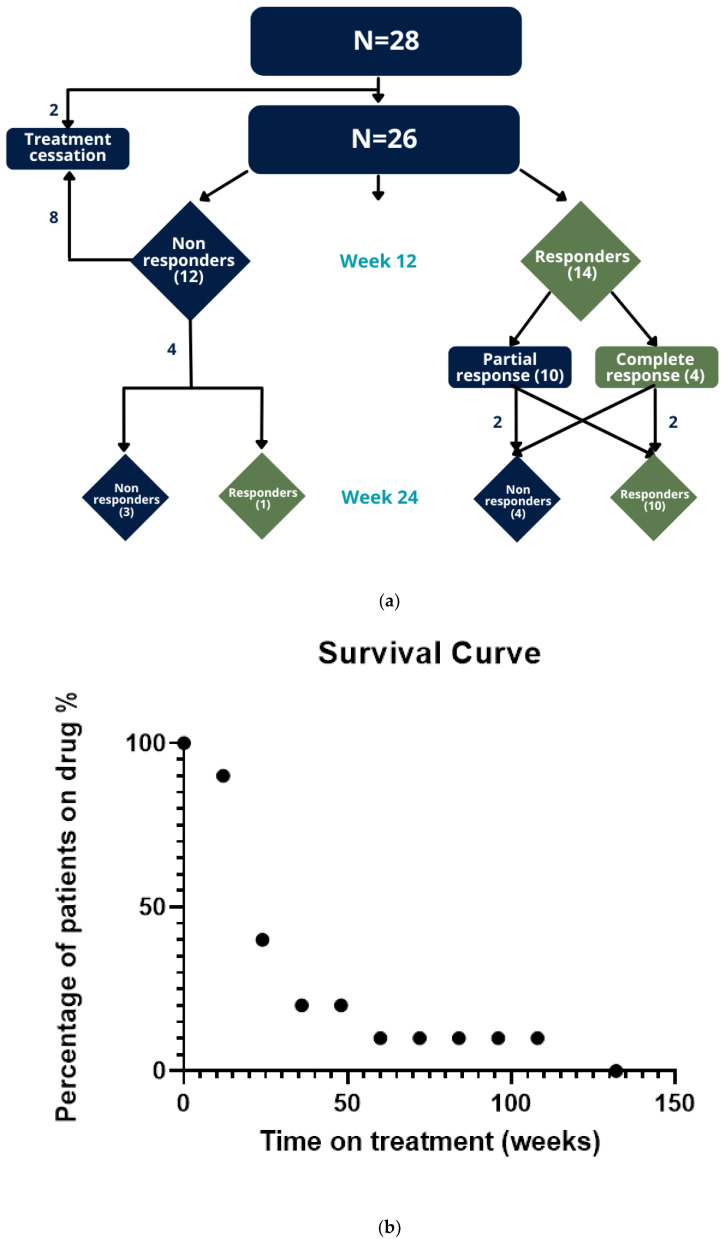
**Methotrexate treatment outcomes.** (**a**) Chart illustrating MTX treatment response at week 12 and week 24. (**b**) MTX survival curve across a follow-up of 144 weeks.

MTX Treatment and Outcome. Treatment and treatment outcomes are presented in Table 2. Patients started MTX treatment at an average age of 2.7 years (SD = 1.2). The average dose administered during the treatment period was 0.45 mg/kg (SD = 0.09). Median MTX treatment duration was 5 months (IQR = 5.5). After 12 weeks of MTX treatment, a significant IGA improvement was demonstrated across the entire cohort (mean ranks = 0.98, *p* = 0.00032). Among 28 patients, we detected a response rate of 50.0%: 12 patients (42.8%) had no response, 10 patients (35.7%) exhibited partial response (achieved a 50% decline in their IGA), and 4 patients (14.2%) showed complete/almost complete response (reached IGA 0/1) (Figure 1a). At the 24-week follow-up, 18 patients were assessed for treatment efficacy. Among them, the response rate was 61.1%: seven patients (38.8%) had no response, five patients (27.7%) showed partial response, and six patients (33.3%) had complete/almost complete response. Of the 14 patients who responded at week 12 and continued MTX therapy, 10 individuals (71.4%) demonstrated sustained improvement at week 24. Conversely, three of four patients who did not respond at week 12 showed no response at week 24.

**Figure 2:**
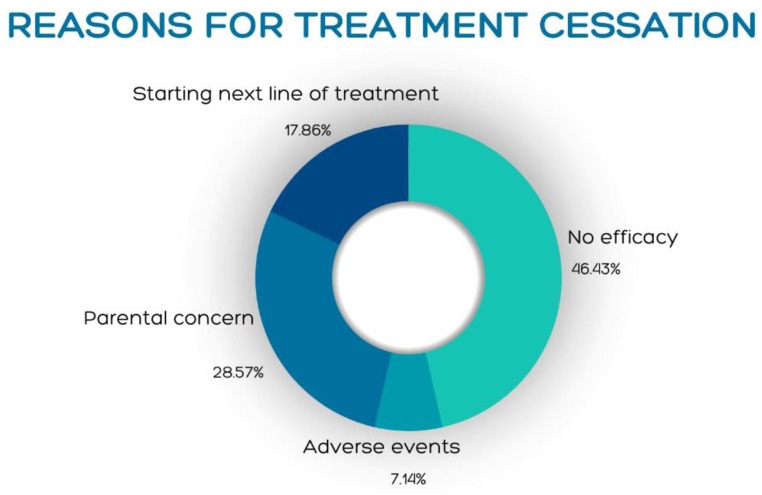
Reasons for cessation of methotrexate therapy.

A survival curve analysis (Figure 1b) shows a gradual decline in patient count from 28 (100%) patients at the beginning of the study to 40% at week 24 and 10% at week 60. Reasons for treatment cessation are presented in Figure 2 and most commonly included loss/lack of efficacy (46.43%) and parental concern (28.5%).

During the MTX treatment course, 16 (57.1) patients reported adverse effects. Seven patients reported mild gastrointestinal symptoms and three patients had elevated liver enzymes. Two patients (7.1%) developed anemia and one experienced Kaposi varicelliform eruption. Only two patients (7.1%) discontinued MTX due to adverse events.

Following methotrexate cessation, patients were frequently treated with dupilumab (57.6%) and topical steroids (42.3%) (Table 2).

## 4. Discussion

Our study presents a cohort of infants and toddlers younger than four years old, with moderate to severe AD who were treated with MTX. 

We found moderate efficacy for MTX treatment: at 12 weeks, 50.0% of the patients presented 50% improvement in their initial IGA score, and among them 10% achieved IGA 0/1. At 24 weeks, response rates were similar, with a greater number of patients presenting a complete response. Our results are comparable with previous studies involving older pediatric patients with AD treated with MTX. These studies reported response rates of 53–75% and a 1.3–1.4 unit decrease in the investigator global assessment (IGA) score [23,24] or a 25.02 ± 8.21 reduction in the severity scoring for atopic dermatitis (SCORAD) [19] 3–5 months after MTX treatment. Response sustained 6–9 months after treatment initiation [20,25]. Among initially responsive patients at week 12 who continued treatment, the majority maintained and improved their response by week 24, suggesting a high likelihood of sustained response. In contrast, non-responders at week 12 showed no response at week 24; however, the small sample size limits definitive conclusions. These findings are consistent with previous reports indicating the expected response to MTX within 6–12 weeks [11,26]. The yield of continuing MTX beyond week 12 without a response is questionable.

Since information regarding doses and monitoring in infants and toddlers with AD is sparse, we compared our data with the consensus treatment guidelines for MTX in pediatric patients with inflammatory skin diseases [27]. Nevertheless, this consensus does not provide specific dosage recommendations for AD in pediatric patients, and a starting dosage ranging from 0.3 to 0.5 mg/kg was recommended along with a maintenance dose of less than 1 mg/kg/week. Other studies in pediatric AD patients reported dosages of 0.2–0.7 mg/kg [25,26]. In our study, patients were treated with an average dosage of 0.46 mg/kg per week. Monitoring of the complete blood count (CBC), liver function, and kidney function in our study was in line with the consensus guidelines, which recommend monitoring after the first month of treatment, followed by monitoring every three to four months.

Our results support a good safety profile for pediatric patients younger than four years old treated with MTX. Only two patients (7.1%) discontinued treatment due to adverse events (anemia and impaired liver function tests) which resolved following drug cessation. These findings are consistent with prior investigations showing a favorable safety profile for MTX in older children [16,17,18,23,25,26,28,29].

Upon follow-up, 57.6% of patients, who discontinued MTX, initiated treatment with dupilumab (Table 2). With the recent approval of dupilumab in pediatric patients above the age of 6 months [30], and considering its efficacy and safety profile, MTX may be considered a feasible alternative therapeutic option. This is particularly relevant in situations where dupilumab is either inaccessible or prohibitively expensive, or in cases where insurance providers mandate prior treatment trials before granting approval for its utilization.

Our study elucidated a significant discrepancy observed in young pediatric patients treated with MTX. Although MTX demonstrated moderate efficacy and a favorable safety profile with mild side effects treatment, dropout was prominent, surpassing previous reports [31,32]. This was attributed mostly to inadequate perceived efficacy (46.43%) and parental concern (28.5%). The discrepancy raises questions regarding the factors influencing treatment decisions and outcomes in this particular patient population. It is plausible that perceived moderate efficacy can be partially attributed to initial mild improvement during treatment, as many patients discontinued treatment due to parental concerns before the full efficacy of the intervention could be appreciated. It is also plausible that in the context of a younger age group, parental concerns outweigh the perceived mild improvement in AD scores. This discrepancy emphasizes the intricate interplay between subjective experiences, patient satisfaction, and objective clinical assessments, warranting further exploration.

Our study has several limitations, which should be acknowledged. Its retrospective design may introduce incomplete or biased data. The lack of a control group limits the ability to establish causality and make direct comparisons. The small cohort size (*n* = 28) reduces generalizability and increases the risk of sampling biases.

In conclusion, our study examines the use of MTX in young pediatric patients with moderate to severe AD, showing a good safety profile and moderate efficacy, which might be underestimated due to parental concern and early treatment termination. Our results support the use of MTX as an off-label therapeutic option for this age group. Future studies with larger sample sizes, prospective designs, randomized controlled trials, and longer follow-up periods are needed for more robust evidence on MTX treatment in pediatric AD.

## Figures and Tables

**Table 1:** Patient demographic and clinical characteristics.

Characteristics		All Patients *n* = 28
Sex *n* (%)	Female	11 (39.3%)
	Male	17 (60.7%)
Age at onset of AD, years median, (IQR)		0.4 (0.9)
Personal history of asthma/food allergy		12 (42.8%)
Family history of atopy	Yes	16 (57.2%)
Hospitalizations due to AD prior to MTX treatment	No	23 (82.1%)
	Yes	4(17.8%)
	*n* = 27	
Treatments prior to MTX initiation, *n* (%)	Topical corticosteroid	27 (96.4%)
	Topical phosphodiesterase 4	2 (7.1%)
	Topical calcineurin inhibitors	19 (67.8%)
	Systemic corticosteroid	4 (14.2%)
	NBUVB	10 (35.7%)
	Climotherapy	2 (7.1%)

**Table 2:** MTX treatment details and outcomes.

Characteristics	All Patients N = 28	
Age at MTX initiation, years mean (SD)		2.7 (1.2)
Weight, kg		13.7 (2.9)
MTX initiation dose mg/kg, mean (SD)		0.45 (0.09)
IGA at MTX initiation, mean (SD)		3.78 (0.4)
IGA after 12 weeks of MTX treatment, mean (SD)	*n* = 26	2.85 (0.9)
IGA after 24 weeks of MTX treatment, mean (SD)	*n* = 18	2.37 (1.0)
Treatment duration, months, median (IQR)		5 (5.5)
Response at 12 weeks	Stopped prior to week 12	2
	No response	12
	Partial response	10
	Complete/almost complete response	4
	*n*	28
Response at 24 weeks		
	No response	7
	Partial response	5
	Complete/almost complete response	6
	*n*	18
Adverse events (%)	None	13 (42.8)
	Gastrointestinal	7 (25)
	LFT elevation	3 (10.7)
	Anemia	2 (7.1)
	Joint pain	1 (3.5)
	Weakness	1 (3.5)
	Kaposi varicelliform eruption	1 (3.5)
Treatments after discontinuing MTX		
	Topical steroids	11 (42.3)
	Dupilumab	15 (57.6)
	Phototherapy	2 (7.6)
	Cyclosporin	2 (7.6)
	Topica CNI	3 (11.5)
	Systemic steroids	1 (3.8)
	No treatment	1 (3.8)
	Loss to follow up	2 (7.6)

## Data Availability

Data are available on request with restrictions (e.g., privacy or ethical). The data presented in this study are available on request from the corresponding author. The data are not publicly available due to the hospital’s policy.
